# HPV infection and vaccination in Systemic Lupus Erythematosus patients: what we really should know

**DOI:** 10.1186/s12969-016-0072-x

**Published:** 2016-03-08

**Authors:** Ingrid Herta Rotstein Grein, Noortje Groot, Marcela Ignacchiti Lacerda, Nico Wulffraat, Gecilmara Pileggi

**Affiliations:** Department of Pediatric Immunology and Rheumatology, Wilhelmina Children’s Hospital, University Medical Centre Utrecht, Room number KC 03.063.0, PO BOX 85090, 3508 AB Utrecht, The Netherlands; Department of Pediatric Rheumatology, Pequeno Príncipe Hospital, Curitiba, Brazil; Department of Pediatric Immunology, Sophia Children’s Hospital - Erasmus MC, Rotterdam, The Netherlands; Department of Gynecology, University of the Estate of Rio de Janeiro, Rio de Janeiro, Brazil; Department of Pediatric, Division of Immunology and Rheumatology, School of Medicine of Ribeirão Preto - University of São Paulo, São Paulo, Brazil

**Keywords:** Human papillomavirus infection, HPV, Systemic lupus erythematosus, SLE, Immunosuppression, Cervical dysplasia, Cervical cancer, Vaccines

## Abstract

Patients with Systemic Lupus Erythematosus (SLE) are at increased risk for infections. Vaccination is a powerful tool to prevent infections, even in immunocompromised patients. Most non-live vaccines are immunogenic and safe in patients with SLE, even if antibody titres are frequently lower than those of healthy controls. Human papillomavirus (HPV) infections are more prevalent in SLE patients when compared to the healthy population. Low-risk types of this virus cause anogenital warts, while high risk types are strongly related to pre-malignant cervical abnormalities and cervical cancer. HPV vaccines have been developed to prevent these conditions. Although little is known about HPV vaccination in SLE, few studies in patients with autoimmune rheumatic diseases (AIRDs) have shown that HPV vaccines are safe, and capable to induce an immunogenic response in this group of patients. To date, available data suggest that HPV vaccines can be given safely to SLE patients. Given the increased incidence of cervical abnormalities due to HPV in SLE patients, this vaccination should be encouraged.

## Background

Systemic lupus erythematosus (SLE) is an immune disorder that predominantly affects women of reproductive age. The disease is characterized by chronic inflammation, circulating autoantibodies, and heterogeneous multisystemic involvement. It is well known that SLE patients are at increased risk for infections [1–7]. This might be due to the dysregulation of their immune system as well as the immunomodulatory therapy used by these patients [2, 4, 7–10].

Vaccines present one of the most effective tools to prevent infectious diseases. Ideally, vaccines should be immunogenic (i.e., capable to induce an antibody response), effective (i.e., provide protection) and safe. Vaccine safety in patients with chronic inflammatory conditions can be broken down into two separate concerns. Firstly, there is a potential risk of causing an exacerbation of the underlying disease. Secondly, in case of live-attenuated vaccines, there is a possibility to induce overt infection [9, 11]. When considering vaccinating a SLE patient, these issues should be addressed [2, 4, 8–11].

Protective immunity is dependent on a functional immune system that induces both adequate antibody levels as well as memory to achieve short term and long-term protection [2, 8, 9, 11]. As SLE patients have a dysfunctional immune system, there is a risk that their immune response to vaccination could be impaired. However, studies have demonstrated that most vaccines are efficacious in patients with SLE, even if specific immune responses may be reduced when compared to the responses of the healthy population [4, 5, 11–13].

In this review, we aim to give an overview of the risks of contracting oncogenic HPV infections in patients with SLE. Additionally, we describe the importance and relevance of the HPV vaccines in this group of patients. Three non-live protein subunit vaccines for HPV were approved in the last decade: bivalent (bHPV), quadrivalent (qHPV), and most recently, 9-valent (9vHPV) vaccines [14–16]. These vaccines were developed to prevent pre-malignant cervical lesions and cervical cancer. qHPV and 9vHPV also prevent benign conditions caused by HPV, like anogenital warts. All these conditions are more common in patients with SLE when compared to healthy population [17–27].

## Vaccination in patients with Systemic Lupus Erythematosus

The European League Against Rheumatism (EULAR) has published general recommendations for the use of vaccines in autoimmune rheumatic diseases (AIRDs) [28, 29]. When indicated in national guidelines, non-live vaccines can be administered independent of medication use. It is recommended to withhold live-attenuated vaccines in patients on high-dose disease modifying anti-rheumatic drugs (DMARDs), high-dose glucocorticosteroids or biological agents. Boosters vaccinations can be considered in patients receiving a low dose of immunosuppressive medications [28, 29].

Irrespective of the AIRD, the majority of immunosuppressive drugs do not seem to have a detrimental effect on the immune response against vaccines, especially when the drug is administered in low doses [28, 29]. There is one important exception: rituximab, an anti CD-20 antibody. This drug acts through depletion of lymphocytes B and therefore strongly impairs the humoral immune response to vaccines. For this reason, it is recommended to vaccinate before or at least six months after administration of this medication [11, 28, 29].

Pneumococcal polysaccharide vaccine (PPSV23) and influenza vaccines have been researched extensively in patients with SLE, as these pathogens are responsible for the most common infections in patients with SLE [1, 4, 5]. Studies show that these two non-live composite vaccines are safe in this patient population. Vaccinated patients show hardly any change in disease activity. Patients did have a lower specific immune response when compared to the general population, but the majority of them produced protective antibody levels. No clear effect of immunosuppressive drugs or biological agents on the antibody titres was reported [1, 4, 5]. In pediatric patients, only influenza vaccines have been studied, with similar results to the adults trials [1, 11, 30–33].

The Hepatitis B Virus (HBV) vaccine, another non-live composite vaccine, has been studied in this population as well. One adult study with 28 patients and one pediatric study with 64 patients had showed lower antibody seroconversion rates, but still adequate humoral response against the virus in patients with SLE. There were no significant exacerbation of disease, nor adverse effects after HBV vaccination [1, 11–13].

The most important concern regarding live-attenuated vaccines in patients with SLE relates to the risk of developing overt infections of live-attenuated pathogens in the vaccine [3]. Only two prospective studies using a live attenuated virus vaccine in SLE patients have been published. One studied the varicella-zoster virus (VZV) vaccine in childhood-onset SLE patients who were previously immune to varicella virus. This study showed adequate immunogenicity and safety [34]. The other also studied the VZV-vaccine, in 10 SLE patients older than 50 years. The vaccine was safe: no episodes of herpes zoster or vesicular rash, no serious adverse events or SLE flares were reported [6].

Vaccination in immunocompromised patients can reduce the burden of infections [11, 28]. As these patients have many benefits from effective vaccination strategies, new vaccines should be assessed in this population as well. The most recent example are vaccines against the human papillomavirus (HPV), that have been studied in healthy and immunocompromised individuals in the last years, with satisfactory results.

## Human Papillomavirus infections and vaccination in the healthy population

HPV infection is the most common sexually transmitted disease (STD) in the world [35–39]. The virus is able to infect the skin, mucous membranes of the anogenital region, as well as conjunctiva, oral and nasal mucosas, larynx and pharynx [37, 40]. Depending on the subtype, the virus can cause pre-malignant cervical abnormalities and cervical cancer as well as benign conditions such as anogenital warts. Besides sexual transmission, there are studies reporting transplacental and perinatal transmission [41–43]. It seems that the transmission is significantly lower when caesarean section is performed. In the majority of cases, the viral DNA does not persist in the newborn for more than 6 months [41–45]. Recurrent laryngeal papillomatosis, which is the most important clinical consequence of infantile HPV 6 and 11 infections, is a rare consequence of HPV chronicity in the newborn [44, 45].

The prevalence of HPV infection varies according to gender, age, population and geographic region. A large systematic review about HPV prevalence in women encompassed data from 70 countries worldwide [46]. Age is the strongest predictor, as the highest HPV prevalence in all studies around the world is found in women between 16 and 24 years old [35–37, 39, 46]. The review showed an age-stratified HPV prevalence that varied considerably across geographical regions and study population, ranging from less than 2 % to approximately 70 %, even in the same continent. Women under 25 were the most affected group in all study populations. Some regions, especially in America and Africa, showed a second peak among women older than 45 years old [46].

HPV is a DNA virus from the papillomavirus family, which covers over 120 genotypes classified according to the organization of DNA sequences and their oncogenic potential. About 40 of them are able to infect the human anogenital tract [35–38]. The most important types are showed in Table 1. The low risk subtypes are responsible for benign abnormalities such as anogenital warts. The subtypes 16 and 18 are responsible for 70 % of all cervical cancers, around 50 % of high-grade intraepithelial lesions (HSIL) and 30–50 % of low-grade intraepithelial lesions (LSIL). Together with the other high risk subtypes, they encompass 99*.*7 % of all cervical dysplasias, pre-malignant abnormalities, and cervical cancers [35–38]. A system for reporting these cervical cytologic diagnoses is the Bethesda System, created in 1988 and last updated in 2014 (Table 2) [47, 48].

**Table 1:** Classification of HPV genotypes by cervical oncogenicity

Oncogenic potential	HPV genotypes
Low risk	6, 11, 40, 42, 43, 44, 54, 61, 72, 81, 89
High risk	16, 18, 31, 33, 35, 39, 45, 51, 52, 56, 58, 59
Probably/Possible high risk	26, 53, 66, 68, 73, 82

*Abbreviations*: *HPV* human papillomavirus

Adapted from Erickson et al. [37]

**Table 2:** Bethesda System (Last update in 2014)

Terminology	Interpretation
Negative for intraepithelial lesion or malignancy	No cellular evidence of neoplasia
Squamous cells:	
Atypical squamous cells:	
Of undetermined significance (ASC-US)	Undetermined
Cannot exclude HSIL (ASH-H)	Cannot exclude pre-malignant lesion
Low-grade squamous intraepithelial lesion (LSIL)	HPV/mild dysplasia/CIN-1
High-grade squamous intraepithelial lesion (HSIL)	Moderate and severe dysplasia/CIN-2 and CIN-3/SIC
Squamous cell carcinoma	Invasive cancer
Glandular cells:	
Atypical glandular cells	Undetermined or cannot exclude pre-malignant lesion
Endocervical adenocarcinoma is situ	CIS
Adenocarcinoma	Invasive cancer

*Abbreviations*: *CIN* cervical intraepithelial neoplasia, *CIS* carcinoma in situ, *HPV* human papillomavirus

Adapted from Nayar and Wilbu [48]

Cancers caused by HPV include all cancers of the cervix, most anal (88 %) and vaginal (70 %) cancers, and part of vulvar (43 %), penile (50 %), and oropharyngeal cancers (26 %) [40]. The most recent estimate of the worldwide incidence and mortality of 27 major types of cancer, ranked cervical cancer as the fourth most common cancer in women. Interestingly, 84 % of the cases as well as 87 % of the deaths due to cervical cancer occurred in the more poorly developed regions [40, 49]. This is a consequence of more effective programs of population screening in developed countries, which provide early diagnosis and treatment and a higher chance of curing this cancer [40]. In the absence of screening programmes as in poorer countries is often the case, simple vaccination strategies could thus be extra effective.

**Fig. 1:**
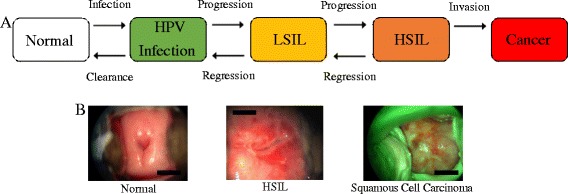
**a** Sequential steps for cervical cancer: Infection with carcinogenic HPV type(s), followed by detectable viral persistence, linked to the progression of cervical premalignant lesion, and finally, invasion (Adapted from Schiffman M. et al.) [50]. **b** Colposcopy of normal cervix, high-grade squamous intraepithelial lesion (HSIL) and Squamous Cell Carcinoma

HPV infection alone is not sufficient to develop cervical abnormalities. The combination of the virus’ oncogenicity, its persistence in the infected tissue and the response of the host immune system determine the evolution of the infection [35–38, 40, 50]. In a large number of cases, the immune system is capable of clearing the virus in 1 to 5 years, without any treatment. Thus, only a minority of individuals infected with HPV develop clinical complications (Fig. 1). Women who are younger at first sexual intercourse or who have multiple sexual partners have a higher risk to develop HPV abnormalities, since they are more likely to be exposed to oncogenic subtypes as well as to a high charge of the virus. Smoking and hormonal contraception can cause immunological dysregulations that facilitate the genetic expression of HPV. The presence of other STDs increases the risk of disease development as well. In addition, immunosuppression or immunodeficiency can facilitate viral persistence in the host [35–38, 40, 50].

As the evolution of HPV infection to pre-malignant cervical abnormalities and cervical cancer takes decades in health women, it is possible to identify pre-malignant lesions by conducting screening tests. Conventional cytology tests (Papanicolaou test or Pap test) are the most widespread method used in cervical cancer screening. The test should be performed at each 3-year interval [16, 50–52]. In the past years, HPV DNA tests have been incorporated by developed countries for the screening of HPV infections. These tests have a higher sensitivity and are slightly less specific when compared with Pap test. The new guidelines suggest that the cervical screening of women 30 and over can be extended to 5-year interval when negative Pap test is combined with HPV DNA test [16, 50–52]. For women younger than 30 years, HPV DNA testing alone or in combination with Pap test are not recommended due to the high prevalence of HPV infection at this age, as well as the rarity of progression of those infections to invasive cancer [52].

Evidence from current studies suggests that the periodic tests should be complemented with HPV vaccination, aiming at primary prevention of infection with the virus [16, 40, 51]. Currently, three types of prophylactic HPV vaccines are available. The bHPV vaccine is directed against two high-risk genotypes, 16 and 18. The qHPV vaccine is also directed against these two high-risk HPV genotypes, but also provides protection against the low risk genotypes 6 and 11. Clinical data shows that both vaccines are safe and generate adequate antibody levels up to 4.5 years after vaccination [53–56]. A 9-valent HPV vaccine was licensed in December 2014 by the Food and Drug Administration (FDA), and in March 2015 by the European Medicine Agency. This vaccine offers protection against an additional five HPV high-risk genotypes. This should increase prevention of cervical cancer from 70 to 90 %, as well as prevent 85–95 % of HPV-related vulvar, vaginal and anal cancers. As this vaccine has just been approved, no clinical effectiveness data is yet available [14, 15, 57, 58].

The HPV vaccines have been incorporated in National Immunization Programs (NIP) across the globe, targeting 9 to 13 years old girls, with an original three dose regimen [40, 59]. Recently, the World Health Organization (WHO) updated its HPV vaccines position paper to recommend a two dose regimen with a 6-month interval between doses for immunocompetent girls younger than 15 years at the time of the first dose [59]. Different from European countries, The United States, Canada and Australia have incorpored HPV vaccination for boys from 9 to 12 years old in their NIP [14, 40]. Women younger than 26 years and men younger than 21 years can also be benefited by the HPV vaccination [14, 40, 59].

**Fig. 2:**
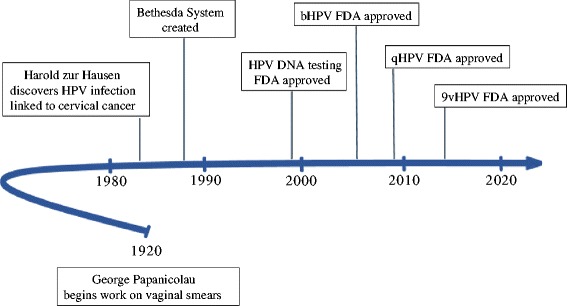
Important events regarding cervical cancer and HPV. Abbreviations: bHPV, bivalent vaccine; qHPV, quadrivalent vaccine; 9vHPV, 9-valent vaccine; HPV, human papillomavirus; FDA, Food and Drug Administration. Adapted from Lees et al. [16]

All three vaccines show high efficacy in prevention of vaccine-specific HPV-type infection and associated high-grade cervical dysplasia in HPV-naïve women (Table 3) [57]. Despite bHPV and qHPV vaccines having been approved only 10 years ago, a meta-analysis of 20 studies performed in developed countries has already showed significant decrease of HPV prevalence in young vaccinated men and women [60]. Due to the lag-time between HPV infection and the development of cancer, a decreased incidence of HPV-related cancers can only be determined after several decades. In order to reduce the incidence and mortality from cervical cancer globally, these vaccines should have a high coverage in the population [40]. Figure 2 shows the most important events in the history regarding the screening and prevention of HPV infection and cervical cancer.

**Table 3:** HPV vaccines efficacy and cervical cancer coverage

Vaccine	HPV genotypes	Vaccine efficacy	Cervical cancer coverage
bHPV	6 and 18	>98 % for HPV disease related to genotypes 6 and 18	70 %
qHPV	6, 11, 16 and 18	>99 % for HPV disease related to genotypes 6, 11, 16 and 18 (women) 90 % for external genital disease (men)	70 %
9vHPV	6, 11, 16 and 18 31, 33, 45, 52 and 58	>99 % for HPV disease related to genotypes 6, 11, 16 and 18 96,7 % for HPV disease related to genotypes 31, 33, 45, 52 and 58	90 %

*Abbreviations*: *bHPV* bivalent vaccine, *qHPV* quadrivalent vaccine, *9vHPV* 9-valent vaccine, *HPV* human papillomavirus

Adapted from Committee Opinion number 641 [57]

## Human Papillomavirus infections and vaccination in Systemic Lupus Erythematosus patients

Studies from all over the world showed that HPV infections are more prevalent in patients with SLE [17–27]. Santana et al. performed a systematic review of 33 studies. They concluded that the diagnosis of SLE directly increases the risk of developing cevical dysplasias and pre-malignant lesions [20]. Although the majority of the studies showed that SLE patients had more pre-malignant lesions than healthy women, the prevalence of cervical cancer was similar between SLE patients and healthy populations in almost all studies [20]. Only one study included in the review found an increased prevalence of cervical cancer in SLE patients [20, 21]. The increased risk of pre-malignant lesions in this population was confirmed in a meta-analysis performed by Zard et al. [23]. Seven studies about the prevalence of pre-malignant cervical lesions in patients with SLE were included. They showed a 9-fold increase risk on pre-malignant cervical lesions in this population when compared to healthy individuals [23]. The studies included in this meta-analysis did not include any long term follow-up data. Whether these pre-malignant lesions progress to cervical cancer remains unclear. An explanation for the higher prevalence of cervical dysplasia in SLE patients is that clearance of HPV could be related to adequate innate and adaptative immune responses. As these are impaired in patients with SLE, clearance of the virus could also be impaired or merely delayed, resulting in persistent carriage of HPV, as well as persistent lesions [20, 23, 24, 61].

There is no consensus in the literature regarding the role of immunosuppressive drugs as a predisposing factor for the cervical abnormalities caused by HPV [19, 20, 25]. The majority of the studies showed no association. However, some evidence suggests azathioprine and intravenous cyclophosphamide use could contribute to the development of persistent HPV infections and subsequent complications [19, 20, 25–27].

It is clear that patients with SLE are at increased risk to develop HPV infections. Thus, it is reasonable that more rigorous criteria should be applied to cervical cancer screening in SLE patients, with shorter intervals in between examinations than the general population, associated to HPV vaccination [19]. The introduction of the HPV vaccines presents an opportunity for cervical cancer prevention. SLE patients should benefit from preventive vaccination. Therefore, it is essential that the safety, immunogenicity and efficacy of the HPV vaccine in this specific population will be studied.

The EULAR recommendations for both children and adults with rheumatic diseases state that the HPV vaccine should be considered in patients with autoimmune diseases. An association with venous thromboembolic events (VTE) and the qHPV vaccine had been reported, which led to a side note on this in the recommendations regarding the vaccine [62]. However, current evidence from 997.585 girls did not show any association between HPV vaccination and possible VTE. In this large cohort, no associations between HVP vaccination and autoimmune or neurological adverse events could be shown either [63].

Since the HPV vaccines were licensed, six original articles and one systematic review have been published regarding the safety and immunogenicity of these vaccines in patients with autoimmune disease [53, 54, 64–67]. Three articles studied the safety and immunogenicity of the vaccine in SLE patients [66–68], two in juvenile idiopathic arthritis (JIA) patients [64, 69], one in patients with juvenile dermatomyositis (JDM) [68] and one in patients with inflammatory bowel disease (IBD) [65]. Overall, the vaccine seems to be safe and immunogenic in patients with an autoimmune disease.

The largest study assessing immunogenicity and safety of the HPV vaccine in patients with SLE included 50 SLE patients and 50 healthy controls between the age of 18 and 35 years. At twelve months after receiving the first vaccination, seroconversion rates in both groups were adequate (Patients vs Controls: HPV 6: 82 % vs 98 %, HPV11: 89 % vs 98 %, HPV16: 95 % vs 98 %, HPV18: 98 % vs 80 %). A lower immunogenicity of the vaccine was associated with the use of low-dose prednisolone or mycophenolate mofetil. Adverse events were not different between patients and controls. No significant difference in rate of flares in the vaccinated group and a control group of unvaccinated SLE patients was found [66].

A pilot study included 6 patients with childhood-onset SLE who were vaccinated in the context of the national vaccination program [68]. The vaccine induced seropositivity for all isotypes in a high proportion of patients, although the geometric mean titres were lower in patients than those in healthy controls. An uncontrolled study in 20 SLE patients also showed a high seropositivity rate for all isotypes [67]. In both studies, disease activity was measured with the SLE Disease Activity Index (SLEDAI). Prior to vaccination, disease activity was low in all patients. During follow-up, disease activity remained low. Ten mild/moderate flares were reported. These flares were generally similar to the flares the patients had in the year prior to vaccination.

One study evaluated the change in hospitalizations for SLE in the United States since the introduction of the HPV vaccine, to assess its safety [70]. In this study, no evidence of an increased amount of hospitalizations for SLE was found since the introduction of the HPV vaccine.

The HPV vaccines have been studied in small groups of patients with autoimmune diseases. In these groups, the vaccines are shown to be immunogenic and safe. As antibody titres are a proxy for protection for most vaccines, larger, controlled studies are needed to compare the antibody titres in patients and in controls. When antibody titres are low, an adequate immunologic memory could still indicate that individuals are adequately protected when they encounter the virus. Therefore, specific memory against HPV also needs to be assessed in patients with SLE. Additionally, long-term studies are necessary to assess the effect of HPV vaccination on the prevalence of persistent high-risk HPV infections in patients with SLE.

## Conclusion

Infections represent the main cause of death in patients with SLE. Vaccination is the most important tool in the prevention of infections, and for this reason it is an important topic to be discussed in order to improve the care of SLE patients. Although some questions still remain, diverse studies have shown the immunogenicity and safety of non-live vaccines in patients with SLE. Therefore, use of these vaccines should be encouraged by health professionals.

Evidence about the immunogenicity and safety of live-attenuated vaccines in SLE patients is still scarce. More studies are needed to elucidate the level of immunosuppression that turns out patients to be at risk from live agents present in the vaccines, as well as the pattern of immune system response and memory production after vaccination.

HPV is essential in the pathophysiology of pre-malignance cervical abnormalities and cervical cancer, conditions that are highly prevalent among women in all the world. Women with SLE present even higher risk of development HPV diseases. SLE predominantly affects women of reproductive age, that is the same group where the occurrence of HPV infection is increased. As the immune system of SLE patients is abnormal, the clearance of the virus is impaired. The result is the persistence of HPV virus in the cervix of SLE patients more often than in healthy women, what leads to higher prevalence of cervical dysplasia and cancer in SLE female population.

Non-live HPV vaccines are safe and able to produce a protective response even in patients with autoimmune diseases. These findings represent a great benefit for public health. Especially in poorer countries with absent screening programs, HPV vaccination strategies should be implemented. Despites the vaccines have been approved only 10 years ago, studies performed in developed countries already showed significant decrease of HPV prevalence in young vaccinated men and women [40, 60]. Future studies will have to be performed to evaluate the reduction in incidence of cervical cancer and other HPV-related cancers after decades of HPV vaccines implementation, as well as the HPV vaccines’ long term efficacy in patients with SLE.

## Abbreviations

9vHPV: 9-valent HPV vaccine
AIRDs: autoimmune rheumatic diseases
bHPV: bivalent HPV vaccine
DMARDs: disease modifying anti-rheumatic drugs
EULAR: European League Against Rheumatism
HBV: Hepatitis B Virus
HPV: human papillomavirus
HSIL: high-grade intraepithelial lesions
IBD: inflammatory bowel disease
JDM: Juvenile dermatomyositis
JIA: juvenile idiopathic arthritis
LSIL: low-grade intraepithelial lesions
PPSV23: pneumococcal polysaccharide vaccine
qHPV: quadrivalent HPV vaccine
SLE: systemic lupus erythematosus
SLEDAI: SLE Disease Activity Index
STD: sexually transmitted disease
VTE: venous thromboembolic events
VZV: varicella-zoster virus
WHO: World Health Organization
